# Exploring How Blood Cell Levels Influence Subjective Tinnitus: A Cross-Sectional Case-Control Study

**DOI:** 10.3390/audiolres15030072

**Published:** 2025-06-18

**Authors:** Stefani Maihoub, Panayiota Mavrogeni, Gábor Dénes Répássy, András Molnár

**Affiliations:** 1 Maihoub ENT Clinic, Aliakmona Street 16, Limassol 3117, Cyprus; stephaniemaihoub@gmail.com; 2 Tóth Ilona Health Service, Clinical Medical Institute, Görgey Artúr Tér 8, 1212 Budapest, Hungary; panayiota.mavrogeni@gmail.com; 3 Department of Otorhinolaryngology and Head and Neck Surgery, Semmelweis University, Szigony u. 36, 1083 Budapest, Hungary; repassy.gabor.denes@semmelweis.hu; 4 Opera Clinic, Protone Audio Kft., Lázár u. 4, 1065 Budapest, Hungary

**Keywords:** subjective tinnitus, blood cell levels, haematological parameters, tinnitus severity

## Abstract

**Objectives:** This study aimed to analyse the haematological parameters in relation to subjective tinnitus. We hypothesise that abnormal haematological findings may correlate with increased severity and chronicity of tinnitus. This research could lead to improved diagnostic methods and more targeted treatments. **Material and Methods:** A total of 439 patients with primary subjective tinnitus and 274 individuals without tinnitus were enrolled. These participants underwent comprehensive laboratory testing, which included haematological parameters. **Results:** When comparing the white blood cell levels between the tinnitus group and the control group, no statistically significant differences were found (*p* = 0.743). Similarly, comparisons of red blood cell levels (*p* = 0.250), haemoglobin levels (*p* = 0.087), and haematocrit levels (*p* = 0.066) also revealed no significant differences. The platelet levels showed no significant difference between the two groups (*p* = 0.782). According to a logistic regression model, lower levels of haemoglobin (*p* = 0.000) and platelets (*p* = 0.000) significantly predicted higher scores on the Tinnitus Handicap Inventory, indicating self-reported tinnitus severity. Furthermore, lower haemoglobin levels were significant predictors (*p* = 0.04) of developing bilateral tinnitus. Using Spearman’s correlation test, a statistically significant negative correlation (*p* = 0.029) was observed between red blood cell levels and the onset of tinnitus. The frequency of tinnitus demonstrated a significant positive correlation with haemoglobin levels (*p* = 0.04) and haematocrit levels (*p* = 0.043). Conversely, platelet levels showed a significant negative correlation with both tinnitus intensity (*p* = 0.002) and the onset of tinnitus (*p* = 0.033). **Conclusions:** While the haematological parameters showed no significant differences between the tinnitus and control groups, further analyses indicated that certain parameters, such as haemoglobin and haematocrit levels, could potentially influence tinnitus, necessitating further investigation.

## 1. Introduction

Approximately 740 million adults worldwide are affected by the persistent sound of tinnitus [1]. Tinnitus is the perception of hearing sounds without any external source, and its causes can vary significantly [2]. Tinnitus can be categorised as either primary or secondary, depending on its underlying cause. The most significant otological causes include earwax build-ups, inflammation of the outer and middle ears, otosclerosis, and Ménière’s disease. It is important to note that a significant amount of tinnitus is linked to sensorineural hearing loss; however, not every case of tinnitus is associated with clinically detectable hearing loss [3]. Furthermore, it must be mentioned that other causes, particularly systemic factors, are often neglected [4]. Recent studies are exploring the complex interactions between tinnitus and various physiological and psychological factors [5]. This ongoing research not only enhances our understanding of what triggers tinnitus but also offers hope for the discovery of new potential biomarkers and improved methods for diagnosing and treating the condition.

The severity of tinnitus varies significantly and is often associated with various comorbidities, such as depression, anxiety, and sleep disturbances, all of which can greatly affect an individual’s quality of life [6]. Additionally, tinnitus has been linked to several body systems, including the brain and cardiovascular system. There is evidence connecting it to metabolic disorders, such as cardiovascular diseases and issues with endocrine and lipid metabolism [7,8,9]. As a result, the complex nature of tinnitus poses challenges for assessment and management, highlighting the need for an integrated approach that takes into account both auditory and non-auditory factors. A particularly promising area of research explores the relationship between tinnitus and haematological parameters. Blood cells can indicate broader physiological processes, such as inflammation and oxidative stress, which are thought to contribute to the onset and progression of tinnitus. Parameters such as red blood cell (RBC) counts, white blood cell (WBC) counts, haemoglobin (Hb) levels, haematocrit (Hct), and platelet counts may provide valuable insights into the systemic factors influencing tinnitus. The relationship between tinnitus and sensorineural hearing loss is significant, whether the hearing loss is clinically detectable or not. Recent studies suggest that inflammation may be a contributing factor to sensorineural hearing loss [10]. Inflammation can lead to neuroplasticity, which is a crucial mechanism in the context of chronic tinnitus [11].

Given the potential effects of inflammation on the development of chronic tinnitus, our study investigates the relationship between inflammation and chronic tinnitus by examining the connection between haematological parameters and tinnitus characteristics. We hypothesise that abnormal haematological findings may correlate with increased severity and chronicity of tinnitus. This research could lead to improved diagnostic methods and more targeted treatments.

## 2. Materials and Methods

### 2.1. Study Design and Population

This study conducted a retrospective cross-sectional analysis of haematological parameters in individuals both with and without tinnitus. A total of 439 participants experiencing primary subjective non-pulsatile tinnitus were included, along with 274 individuals in a control group who did not have tinnitus. The basic characteristics of the study population are summarised in Table 1. All participants provided written informed consent, and the investigation complied with the Declaration of Helsinki. It also received approval from the Hungarian ETT TUKEB (approval number: BM/29864–1/2024).

The inclusion criteria for the tinnitus group specified that participants must have a clinical diagnosis of primary subjective non-pulsatile tinnitus that has persisted for a minimum of two weeks. This duration is considered significant for evaluating pathological tinnitus and for further assessments. This includes cases associated with sensorineural hearing loss. To confirm eligibility criteria, all patients underwent a thorough otorhinolaryngological assessment conducted by an experienced specialist in tinnitus management to rule out secondary causes of tinnitus. Additionally, each patient underwent tympanometry, acoustic reflex testing, and pure-tone audiometry with tinnitus matching. All patients were required to provide the necessary clinical data to participate in this study. The exclusion criteria were as follows: patients with secondary cases of tinnitus (such as Ménière disease, otosclerosis, earwax buildup, acoustic neuroma, etc.), those experiencing any acute infections (including upper airway infections, lower respiratory tract infections, arthritis, odontogenic inflammations, gastrointestinal infections, etc.), chronic inflammatory diseases, autoimmune disorders, currently diagnosed or treated malignancies, previously diagnosed haematological disorders (including anaemia, coagulopathies, haemoglobinopathies, and malignant haematological conditions), neurological disorders including neuroinflammatory conditions, individuals taking medications known to affect blood parameters, and patients with incomplete medical data. Additionally, none of the patients received any treatment for tinnitus prior to providing blood samples.

### 2.2. Laboratory Testing

Participants provided their consent before blood samples were taken after an overnight fast to ensure consistency. Blood was collected in tubes containing EDTA, which acts as an anticoagulant. All tests were performed in the same laboratory to maintain standardised conditions. Haematological parameters, including WBC count, RBC count, Hb, Hct, and platelet levels, were measured using an automated haematology analyser. The results of the laboratory tests were thoroughly reviewed by the examining doctors.

### 2.3. Audiological Examinations

Before the audiological examinations, all patients underwent microotoscopy and tympanometry, including acoustic reflex testing, to rule out potential causes of conductive hearing loss. Pure-tone audiometry was conducted using a GSI 61 Clinical Audiometer (Grason Stadler, Inc., Milford, CT, USA) by a qualified audiological assistant in each case. The examinations took place in a soundproof booth. Both air conduction (125–8000 Hz) and bone conduction (250–4000 Hz) were measured, utilising headphones and a mastoid vibrator, respectively. In necessary cases, masked bone conduction measurements were performed. The lowest perceivable intensities were identified in 5 dB increments. Prior to the examinations, the sound stimuli were demonstrated to the patients to ensure they understood what to listen for. Standard manual audiograms were created, and pure-tone averages were calculated.

Tinnitus pitch and intensity matching were performed for each case. The pitch matching process was carried out within a frequency range of 125 to 8000 Hz, starting at 1000 Hz. The patient was asked to indicate whether their tinnitus frequency was lower or higher than the sound stimulus played. The frequency was adjusted until the patient identified the most accurate match. Following this, using the frequency determined through pitch matching, the intensity of the tinnitus was measured in 1 dB increments. The pitch and loudness of tinnitus were then manually marked on the audiograms.

### 2.4. Tinnitus Assessment

Tinnitus severity was assessed using the Tinnitus Handicap Inventory (THI), a well-established questionnaire that evaluates the functional, emotional, and catastrophic impacts of tinnitus. Scores were categorised into different severity levels: ‘normal’ (0–16), ‘mild’ (18–36), ‘moderate’ (38–56), ‘severe’ (58–76), and catastrophic (78–100). The total THI score is calculated by summing the points from the three subscales. Participants also provided information about the onset, frequency, intensity, and laterality (unilateral or bilateral) of their tinnitus symptoms. In this study, the validated Hungarian version of the THI was utilised [12].

### 2.5. Statistical Analysis

All statistical analyses were performed using IBM SPSS version 25 software (IBM Corporation, Armonk, NY, USA). The Shapiro–Wilk test was employed to assess the normal distribution of the data. Due to the non-normal distribution, medians and their interquartile ranges (IQRs) were used for continuous variables, and the Mann–Whitney *U* test was applied to determine statistically significant differences. Additionally, Spearman’s correlation test was conducted to analyse potential correlations between the variables. A multinomial logistic regression model was also utilised. The significance level was consistently set at *p* < 0.05.

## 3. Results

Table 1 summarises the current study population’s essential information.

According to the data presented in Table 1, the onset of tinnitus typically peaks at approximately 50 years of age. Additionally, there is a slight predominance of tinnitus in females. In most cases, the onset refers to chronic symptoms of tinnitus. The non-significant differences in age between the tinnitus group and the control group suggest that both groups are statistically comparable.

**Figure 1:**
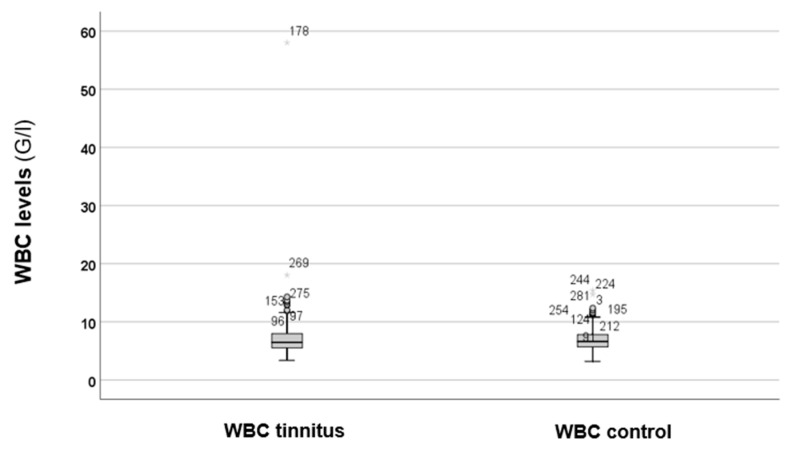
Total WBC levels between the tinnitus and the control groups. In the boxplots, the boxes indicate the interquartile range of the data, while the whiskers show the lower and upper quartiles. The black line that separates the boxes marks the median values. WBC = white blood cell; G = giga; l = litre. The asterisks, circles, and numbers in the figure depict the outliers.

At first, the total WBC counts between the two groups were compared, as shown in Figure 1.

As depicted in Figure 1, there are no obvious differences in the WBC levels between the groups. Statistical analysis using the Mann–Whitney *U* test revealed no statistically significant difference (*p* = 0.743). Therefore, elevated levels of WBC cannot be suspected in cases of tinnitus. Because the results were not significant, we decided against conducting further analysis of the WBC subtypes.

**Figure 2:**
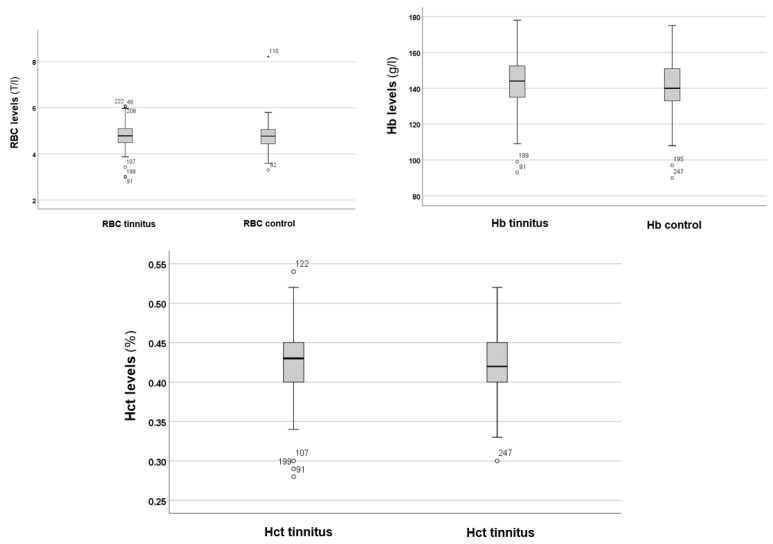
RBC, Hb, and Hct levels in the tinnitus and control groups. In the boxplots, the boxes indicate the interquartile range of the data, while the whiskers show the lower and upper quartiles. The black line that separates the boxes marks the median values. Hb = haemoglobin; Hct = haematocrit; l = litre; RBC = red blood cells; T = tera. The asterisk, circles and numbers in the figure denote the outliers.

In the next step, the RBC, Hb, and Hct levels were analysed, as shown in Figure 2.

Figure 2 shows no significant differences in RBC, Hb, and Hct levels between the tinnitus and control groups. Statistical analysis using the Mann–Whitney *U* test indicated no significant differences in RBC (*p* = 0.250), Hb (*p* = 0.087), and Hct (*p* = 0.066) levels between the tinnitus and control groups. Therefore, alterations in RBC volume and the potential risk for anaemia are not associated with tinnitus.

**Figure 3:**
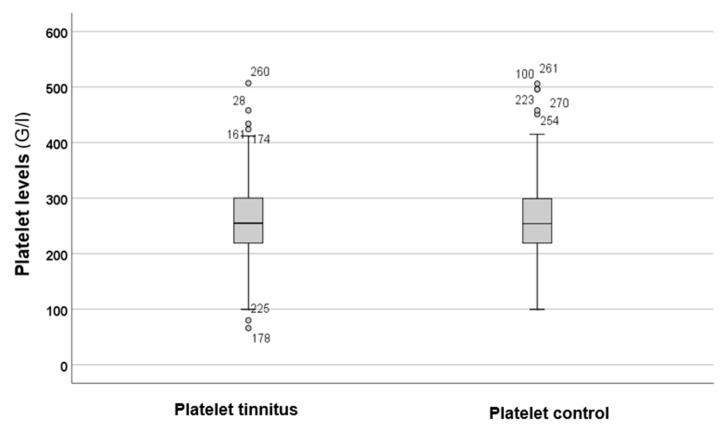
In the boxplots, the boxes indicate the interquartile range of the data, while the whiskers show the lower and upper quartiles. The black line that separates the boxes marks the median values. G = giga; l = litre. The circles and numbers in the figure depict the outliers.

Figure 3 shows the levels of platelets in both the tinnitus group and the control group.

Figure 3 illustrates that there were no differences in platelet levels between the tinnitus and control groups. Statistical analysis using the Mann–Whitney *U* test (*p* = 0.782) indicated that there were no statistically significant differences between the parameters of the two groups.

To further investigate, a multinomial logistic regression model was utilised to analyse the impact of blood cell levels on various tinnitus parameters. The findings are summarised in Table 2.

As shown in Table 2, lower levels of haemoglobin (*p* = 0.000 *; OR: 4,635,442.945, 95% CI = 2,076,573.101–10,347,495.73) and platelets (*p* = 0.000 *; OR: 3,213,479.382, 95% CI = 268,575.428–38,448,974.32) significantly predicted higher total THI scores, which indicate self-reported tinnitus severity. Additionally, lower haemoglobin levels were significant predictors (*p* = 0.04; OR = 0.554, 95% CI = 0.306–1.006) of developing bilateral tinnitus. Pure-tone audiometry averages were not significantly (*p* > 0.05) affected by any parameters; therefore, these calculations were excluded from the table.

**Figure 4:**
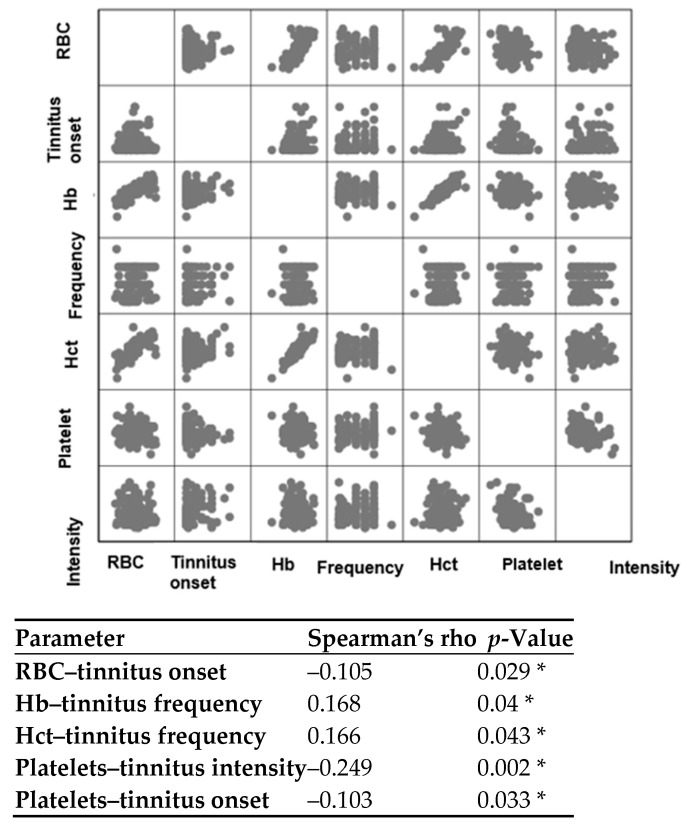
Spearman’s correlation test between various parameters. Hb = haemoglobin; Hct = haematocrit; RBC = red blood cells. The significance level was established at *p* < 0.05, and the asterisk (*) denotes a statistically significant correlation. Please note that only the significant correlations are shown in the figure.

As depicted in Figure 4, a statistically significant (*p* = 0.029 *) negative (rho = –0.105) correlation was observed between RBC levels and the onset of tinnitus. This indicates that as the duration of tinnitus increases, the levels of RBC tend to decrease. In other words, anaemia may be a potential risk factor for experiencing more prolonged tinnitus symptoms. Tinnitus frequencies were significantly correlated with Hb (*p* = 0.04 *) and Hct (*p* = 0.043 *) levels, each showing positive correlations (rho = 0.168 and 0.166, respectively). Platelet levels displayed significant negative correlations with tinnitus intensities (rho = –0.249, *p* = 0.002 *) and the onset of tinnitus (rho = –0.103, *p* = 0.033 *). This suggests that lower levels of platelets may be associated with chronic tinnitus symptoms and greater tinnitus intensities. Pure-tone audiometry average values were correlated with each haematological parameter; however, since no significant correlations (*p* > 0.05) were observed, this analysis was not included in the correlation matrix.

## 4. Discussion

The present study examined the relationship between haematological parameters and subjective tinnitus by comparing baseline blood cell counts in 439 patients with primary tinnitus to 274 controls. Although there were no significant differences in WBC, RBC, Hb, Hct, and platelet levels between the two groups, further analyses identified haematological predictors of tinnitus severity and chronicity. Notably, logistic regression analysis indicated that lower levels of haemoglobin and platelets were significantly associated with higher THI scores and an increased likelihood of experiencing bilateral tinnitus. These findings contribute to the growing research on systemic factors involved in tinnitus pathogenesis and highlight potential biomarkers for clinical assessment.

Our manuscript did not identify any significant differences in WBC levels between the tinnitus and control groups. This finding contradicts the hypothesis that systemic inflammation plays a major role in tinnitus [13]. While some clinical studies and animal models suggest that neuroinflammation may contribute to tinnitus [14], we were unable to establish a direct link between elevated inflammatory markers and chronic tinnitus. This may be due to WBC counts not adequately capturing localised inflammation in the cochlea or neural structures, or it could be attributed to the heterogeneity of tinnitus itself. Among the haematological parameters studied, previous investigations have found a significant relationship only between mean platelet volume and chronic subjective tinnitus. In contrast, other parameters, including WBCs, neutrophils, lymphocytes, platelet count, platelet-to-lymphocyte ratios, neutrophil-to-lymphocyte ratios, red cell distribution width, and platelet distribution width, did not show significant differences when compared to the control group [15]. Previous studies have observed conflicting results regarding cytokine levels in tinnitus. Ozbay et al. conducted a study that found significantly elevated neutrophil-to-lymphocyte ratios in individuals with tinnitus [16]. However, other studies have found that the neutrophil-to-lymphocyte ratio is not a useful parameter for assessing tinnitus [17]. Some previous studies have indicated that CRP levels are not significantly elevated in individuals with tinnitus [18,19]. The study by Weber et al. demonstrated significantly elevated IL-6 levels [20], whereas others did not. In this study, significantly lower levels of IL-10 were found in patients with tinnitus, but not in those with hearing loss [21]. In summary, the relationship between inflammation and tinnitus appears complex, as the existing literature presents conflicting data. Current investigation results suggest that WBCs do not play a significant role in the development of tinnitus.

Despite theoretical connections between anaemia, hypoxia, and tinnitus, we observed no significant differences in RBC count, Hb, or Hct among the groups. This finding contrasts with studies that have linked iron deficiency anaemia to sudden hearing loss and tinnitus [22]. It has been established that the inner ear’s functions depend on oxygen supply [23], and that reactive oxygen species and reperfusion following ischaemia can cause cochlear damage [24]. Previous reports have identified pulsatile tinnitus as a symptom of pernicious anaemia [25]. However, it is important to note that the current investigation did not study cases of pulsatile tinnitus. It is possible that only severe anaemia affects auditory function, while mild to moderate cases, which were prevalent in our study, do not have an impact. Additionally, the presence of compensatory mechanisms may play a role in the cochlea’s inability to adapt to chronic hypoxia, potentially masking the effects of systemic haematological factors [26]. Although the differences in Hb levels between the groups were not significant, logistic regression analysis indicated that lower Hb levels may be risk factors for moderate-to-severe tinnitus and bilateral tinnitus.

Platelet levels did not differ between the groups; however, logistic regression and correlation analyses indicated that lower platelet levels are associated with worsened tinnitus. This is evident in terms of tinnitus handicap, frequency, intensity, and chronicity. This suggests that functional abnormalities in platelets, rather than their count, are significant [27]. Additionally, it supports a nonlinear relationship where extremely low or high counts could be harmful, while mid-range values appear to have a neutral effect. In a previous study, lower platelet levels were found in individuals with subjective tinnitus, suggesting potential autoimmune and inflammatory effects [28]. It is established that inflammation and autoimmune processes can lead to low platelet levels. Since audiovestibular symptoms can occur in various autoimmune diseases [29], it is plausible that tinnitus may have an autoimmune origin. Given the potential connection between platelet levels and tinnitus, further research in this area is necessary. Furthermore, the association of lower haemoglobin and platelet levels with increased tinnitus handicap scores implies that routine haematological screening could provide valuable insights in managing patients with tinnitus. These findings may encourage clinicians to consider evaluating patients’ blood parameters as part of a more comprehensive diagnostic process. Specifically, identifying and managing platelet abnormalities may potentially alter the course of tinnitus, either through direct treatment or by addressing related systemic conditions that contribute to the overall symptom burden.

Several limitations should be acknowledged despite the promising insights presented. The study design limits our ability to establish a causal relationship between haematological changes and the development of tinnitus. Additionally, the exclusion criteria, while necessary to control for confounding factors, may restrict the generalisability of the findings to all patients with tinnitus. Future research should focus on prospective studies involving diverse populations to validate these associations and further investigate how haematological factors influence tinnitus, including longitudinal studies. Moreover, additional specific parameters such as white blood cell subtypes, red blood cell indices, and iron metabolism parameters have not been analysed in detail. Further research should address these issues. Furthermore, exploring interventions that target specific haematological abnormalities could lead to the development of novel therapeutic strategies.

## 5. Conclusions

In summary, although the overall haematological profiles of tinnitus patients and control subjects were similar, certain factors—especially lower levels of haemoglobin and platelets—seem to be associated with increased severity of tinnitus and its bilateral presentation. These findings highlight the importance of considering systemic health and blood-related factors when evaluating and managing tinnitus. Further research is needed to explore these relationships more thoroughly and to assess whether targeted interventions could help reduce the severity of tinnitus.

## Figures and Tables

**Table 1:** The study population’s basic characteristics. IQR = interquartile range; Q1 = first quartile; Q3 = third quartile. * Mann–Whitney *U* test, ** Chi- square test (*p* < 0.05).

	Tinnitus Group (*n* = 439)	Control Group (*n* = 274)	*p*-Value
Age (median years; IQR, Q1–Q3)	51 (25; 40–65)	48 (13.5; 41–54.5)	0.06 *
Sex (men/women)	187/252	116/158	0.81 **
Tinnitus onset (median years; IQR, Q1–Q3)	12 (33; 3–36)		
Tinnitus location			
Right, *n* (%)	110 (25%)		
Left, *n* (%)	138 (31.4%)		
Bilateral, *n* (%)	191 (43.6%)		
Hearing level (median dB; IQR, Q1–Q3)	30 (25; 20–45)		

**Table 2:** A multinomial logistic regression model examined the influence of blood cell levels on tinnitus. CI = confidence interval; OR = odds ratio; Std. = standard; THI = Tinnitus Handicap Inventory. The significance level was set at *p* < 0.05 *. Please note that only the significant results have been included in the table. A tinnitus handicap is considered moderate to severe if the THI score exceeds 38 points.

Dependent	Predictor	*β*	Std. Error	*p*-Value	OR	95% CI (Lower Bound)	95% CI (Upper Bound)
Total THI (moderate to severe handicap)	Haemoglobin	15.349	0.410	0.000 *	4,635,442.945	2,076,573.101	10,347,495.73
	Platelets	14.983	1.266	0.000 *	3,213,479.382	268,575.428	38,448,974.32
Bilateral tinnitus	Haemoglobin	−0.590	0.304	0.04 *	0.554	0.306	1.006

## Data Availability

The data presented in this study are available on request from the corresponding author upon reasonable request.

## Abbreviations

The following abbreviations are used in this manuscript:

CI	confidence interval
CRP	C-reactive protein
EDTA	ethylenediaminetetraacetic acid
G	giga
Hb	haemoglobin
Hct	haematocrit
IBM	International Business Machines Corporation
Il	interleukin
IQR	interquartile range
L	litre
OR	odds ratio
RBC	red blood cell
SPSS	Statistical Package for the Social Sciences
Std.	standard
T	tera
THI	Tinnitus Handicap Inventory
Q1	first quartile
Q3	third quartile
WBC	white blood cell
